# Effects of Low Oxygen Dosages on an Anaerobic Membrane Bioreactor, Simulating the Oxygen Load in an Anaerobic Digester-Dissolved Air Flotation (AD-DAF) System

**DOI:** 10.1021/acsestwater.3c00544

**Published:** 2023-11-11

**Authors:** Antonella L. Piaggio, K.B. Sasidhar, Pravin Khande, Malini Balakrishnan, Jules B. van Lier, Merle K. de Kreuk, Ralph E.F. Lindeboom

**Affiliations:** † Faculty of Civil Engineering and Geosciences, Section Sanitary Engineering, Department of Water Management, Delft University of Technology, Stevinweg 1, 2628 CN Delft, The Netherlands; ‡ School of Civil Engineering, Vellore Institute of Technology, Vellore 632014, India; § Nanotechnology Research, NX Filtration, Josink Esweg 44, 7545 PN Enschede, The Netherlands; ∥ The Energy and Resource Institute (TERI), Darbari Seth Block, IHC Complex, Lodhi Road, New Delhi110003, India

**Keywords:** microaeration, anaerobic membrane bioreactor, wastewater treatment, AD-DAF

## Abstract

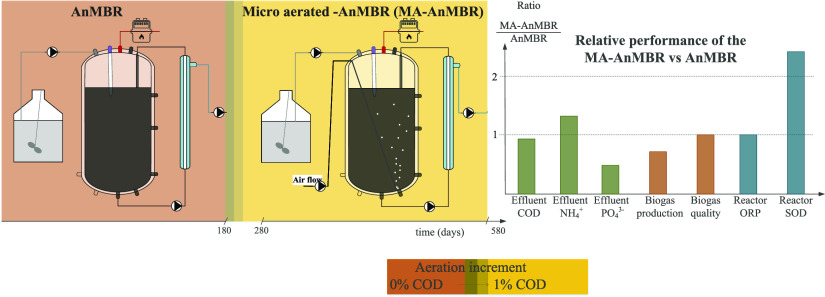

This study reports the effects of microaeration on a laboratory-scale AnMBR (MA-AnMBR) fed with synthetic concentrated domestic sewage. The imposed oxygen load mimics the oxygen load coming from a dissolved air flotation (DAF) unit, establishing an anaerobic digester-DAF (AD-DAF) combination with sludge recycling. Results showed a reduced COD concentration in the MA-AnMBR permeate compared with the AnMBR permeate, from 90 to 74 mgCOD L^–1^, and a concomitant 27% decrease in biogas production. The MA-AnMBR permeate ammonium (NH_4_^+^) concentration increased by 35%, to 740 mgNH_4_^+^-N L^–1^, indicating a rise in the hydrolytic capacity. Furthermore, the MA-AnMBR biomass seemingly adapted to an increased oxygen load, which corresponded to 1% of the influent COD load (approximately 55 mLO_2_ d^–1^). Concomitantly, an increase in the superoxide dismutase activity (SOD) of biomass was detected. Meanwhile, negligible changes were observed in the specific methanogenic activity (SMA) of the microaerated biomass that was subjected to an oxygen load equivalent to 3% of the influent COD load in batch tests. The obtained results showed that an AD-DAF system could be a promising technology for treating concentrated domestic wastewater, improving sewage sludge hydrolysis, and overall organic matter removal when compared to an AnMBR.

## Abstract 2

Micro-aeration of a membrane anaerobic digestor corresponding to 1% of influent COD improves its hydrolytic capacity but decreases biogas production by 27% while keeping the overall performance unchanged.

## 1 Introduction

Anaerobic digestion (AD) is a widely used technology for wastewater treatment due to its low sludge production when compared to aerobic treatment (up to one-tenth), the nutrient-rich effluent, and the production of energy as biogas.^1^ Among the AD technologies, the anaerobic membrane bioreactor (AnMBR) is a promising alternative to treat municipal wastewater from a resource-oriented perspective.^2^ AnMBR units were first developed in the late 1980s for industrial wastewater treatment and are now considered one of the emerging anaerobic technologies that generate high-quality effluents of interest for subsequent reuse.^3^ The principle of an AnMBR is a mixed anaerobic bioreactor connected to a physical membrane separation unit retaining all suspended solids larger than the pore size.

The use of AnMBR for sewage treatment can result in high COD removal and a solids-free permeate, but the technology has considerable constraints linked to the membrane filtration device.^2,4^ The use of membranes to separate solids and liquids is one of the main hydraulic constraints of an AnMBR. Even though the footprint of membrane units is relatively small, a large membrane area is required in municipal wastewater treatment.^4^ Moreover, fluctuations in influent organic loading rate (OLR) and hydraulic flow may negatively impact the sludge filterability and the membrane filtration capacity, decreasing the permeate flux.^5^

Most solids’ physical separation units in wastewater treatment plants are based on screening, flocculation, filtration, adsorption, sedimentation, or flotation.^6^ Among these, dissolved air flotation (DAF) units have a small footprint and are characterized by a high removal of suspended solids under a wide variety of HRTs and OLRs.^7^ When located after a pilot-scale anaerobic digester treating domestic wastewater, DAF removal of suspended solids was reported to reach 96%.^8^ Moreover, previous research showed that a lab-scale DAF could remove up to 95% of the influent total suspended solids (TSS) in the range of 0.03–5.0 g L^–1^, resembling the TSS content of municipal wastewater and the real wastewater from the project target area, the Barapullah drain in New Delhi, India.^9^ Hence, using a different physical separation unit may ensure a high TSS retention while overcoming the AnMBR limitations. Replacing the AnMBR with an AD-DAF system, in which the flotation layer is returned to the anaerobic digester for sewage and drain pretreatment, is proposed. The advantages found in removing the membranes of the system could be jeopardized by the oxygen-saturated flotation layer that may negatively impact the anaerobic conversion process.

Research suggests that exposure of anaerobic biomass to limited amounts of oxygen may only have a negligible impact on strict anaerobes.^10^ Kato et al.^11^ suggested that the tolerance of methanogens to oxygen was mainly due to the activity of facultative bacteria located at the outside of granular consortia in an expanded granular sludge bed reactor (EGSB). Brioukhanov et al.^12^ found high specific superoxide dismutase enzyme (SOD) activities in both methanogens and acetogens, indicating a plausible adaptation to limited oxygen concentrations.

Various authors suggest that microaeration in anaerobic digesters can be beneficial for specific (bio)chemical conversion processes.^10,13,14^ There is no alignment between researchers in what refers to microaeration. Nguyen and Khanal^15^ defined microaeration for systems with oxidation–reduction potential (ORP) between −200 and −300 mV, while Botheju and Bakke^10^ preferred the terminology of *“limited aeration”* to talk about a *process where a certain amount of oxygen is introduced to a basically anaerobic biochemical process*. Limited aeration (below 2% v/v) incorporated in the headspace or liquid phase of a pilot plant digester processing mixed sludge, showed 98% lower hydrogen sulfide concentrations in the biogas with a negligible impact on the methane yield.^16^ Using microaeration in a lab-scale reactor, Lim and Wang^17^ found that the methane yield increased by more than 20% when fed with food waste and concentrated black water. Furthermore, an intermittently microaerated laboratory-scale anaerobic digester CSTR, fed with lignocellulosic feedstock, showed a 50% reduction in volatile fatty acids (VFA) accumulation in comparison to the conditions without microaeration, under an organic loading rate (OLR) of 5 gVS L^–1^ d^–1^.^18^ On the other hand, Botheju et al.^19^ found a negative effect on the methane yield when an oxygen load equivalent to 10% of the influent COD was added to a lab-scale CSTR.

To the authors’ knowledge, no research has been published investigating an AD using a DAF system as a solid retention mechanism, effectively establishing a new anaerobic bioreactor, an AD-DAF system. Due to constraints related to the DAF design loading rates and air bubble sizes in downscaled reactor systems,^20^ laboratory-scale testing of a DAF system is not feasible. Therefore, in our present study, typical DAF oxygen loads were calculated and experimentally simulated in a lab-scale intermittently microaerated AnMBR (MA-AnMBR), fed with synthetic concentrated domestic sewage. The objective of this study was to assess the effect of the given oxygen load on the performance of an MA-AnMBR, mimicking the impact of the compressed air supply in an AD-DAF system needed for flotation. The research focused on the changes in nutrient removal efficiency, especially nitrogen and phosphorus, the overall performance of the MA-AnMBR under various total O_2_ fluxes (in the microaeration range), and the sludge SOD shifts in response to these.

## 2 Methods

### 2.1 Experimental Setup and Tested Influent

**Figure 1:**
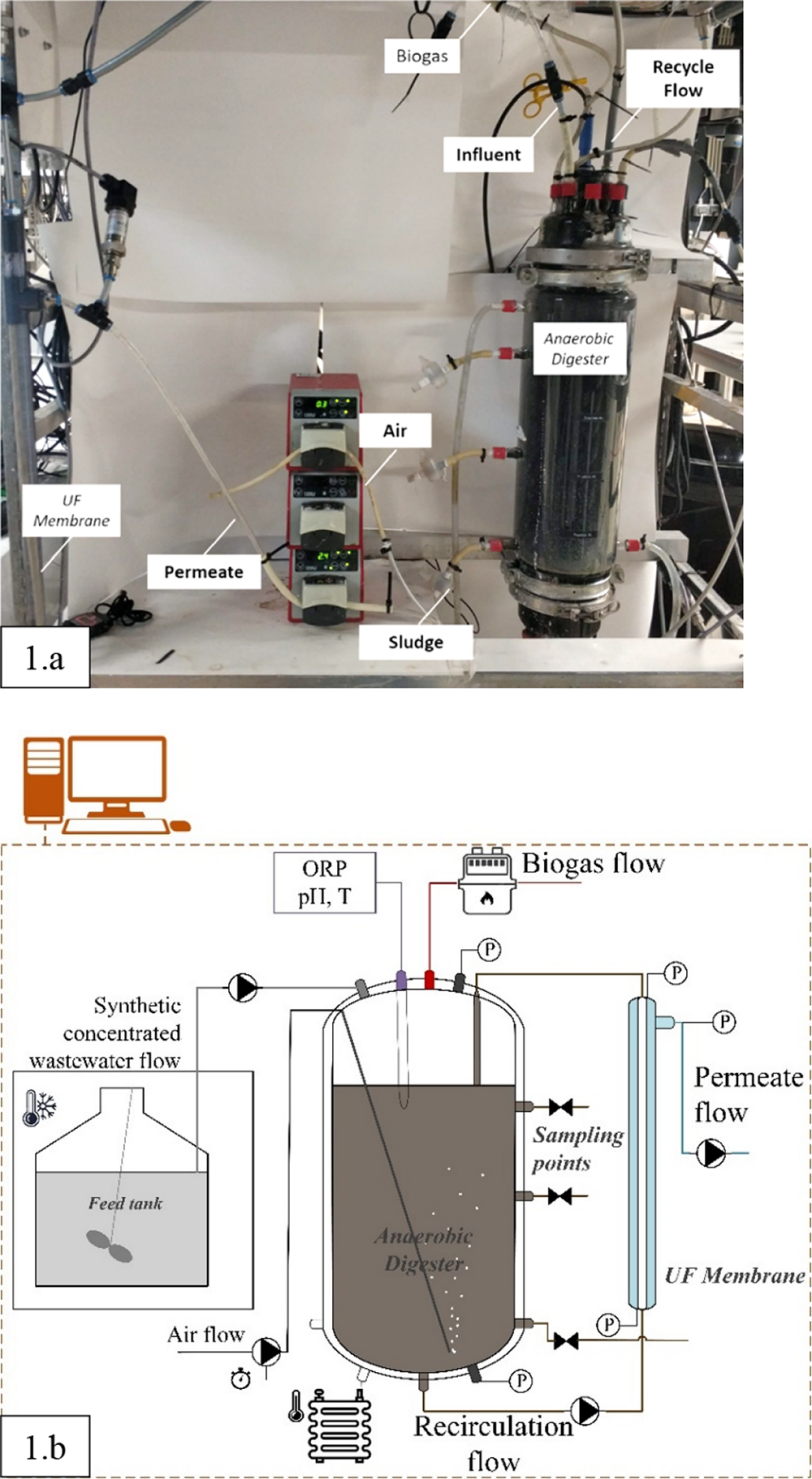
Anaerobic membrane bioreactor (AnMBR) and microaerated AnMBR (MA-AnMBR) set up. Figure 1a shows the laboratory-scale set up, while Figure 1b shows the schematic representation of the laboratory-scale unit.

A laboratory-scale AnMBR was set up to study the effects of microaeration in AD. Here, microaeration was defined as the aeration range at which no significant changes in the oxidation–reduction potential (ORP) were observed in the reactor (below 10%, −450 to −550 mV). The AnMBR consisted of an anaerobic CSTR connected to a side stream inside-out tubular ultrafiltration PVDF membrane, with a pore size of 30 nm (Helyx, Pentair, Minnesota, United States), inner diameter of 5.2 mm, and 640 mm length. The CSTR had a liquid volume of 6.5 and 1.5 L of headspace. The AnMBR was equipped with feed, permeate extraction, aeration, and recirculation pumps (Watson Marlow 120U and 323U, Falmouth, United Kingdom). Reactor ORP, pH, and temperature were continuously measured with a Memosens CPS16D instrument (Endress+Hauser, Reinach, Switzerland). The operational conditions of the AnMBR are described in Table 1, and the reactor setup and scheme can be seen in Figure 1.

**Table 1:** AnMBR Operational Conditions

	unit	value
feed flow	L d^–1^	2.5
permeate flow	L d^–1^	2.3
working volume	L	6.5
temperature	°C	37
hydraulic retention time	d	2.6
solids retention time	d	28
organic loading rate	gCOD L^–1^ d^–1^	1.9
recirculation flow	L d^–1^	1300
cross-flow velocity	m s^–1^	0.6
membrane flux	LMH	10.0

AnMBR and MA-AnMBR were operated under similar conditions, but MA-AnMBR had intermittent air flow. The aeration was introduced in the liquid phase of the anaerobic digester via intermittent cycles of four hours of aeration, followed by four consecutive hours of no aeration. The oxygen load corresponded to an oxygen-overinfluent COD rate of 1.0% (around 55 mLO_2_ d^–1^). Details on the calculation of added oxygen to the AnMBR, to mimic the coupling of an AD-DAF are presented in Supporting Information A.

The synthetic influent composition was adapted from Ozgun et al.^5^ and adjusted to an average COD of 5.2 ± 0.6 g L^–1^, 60 ± 9 mgPO_4_^3–^-P L^–1^ of phosphate, and 249 ± 54 mgNH_4_^+^-N L^–1^ ammonium concentration. Feed composition is further detailed in Supporting Information B. The AnMBR was inoculated with approximately 3.5 L of sludge from a pilot-scale blackwater anaerobic reactor located at the NIOO-KNAW facilities (Wageningen, Netherlands). The sludge had a COD of 43.7 ± 3.4 gCOD L^–1^, total suspended solids (TSS) of 45.8 ± 0.9 gTSS L^–1^ and volatile suspended solids (VSS) of 36.0 ± 1.2 gVSS L^–1^.^21^

#### 2.1.1 Reactor Periods

**Figure 2:**
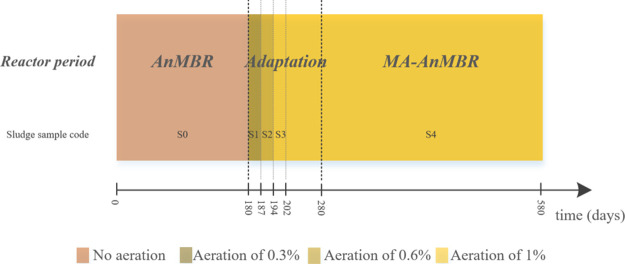
Schematic representation of the reactor periods.

In the first operational period, the AnMBR was operated under complete anaerobic conditions for 180 days (referred to as “AnMBR state”). Sludge extracted from this period was named S0. Microaeration of the AnMBR started afterward and was performed in steps to acclimate the biomass to the aeration dose. Based on the AD-DAF mass balance, the given final daily aeration was calculated to be 1.0% oxygen in comparison to the total COD load, considering an air oxygen content of 21% at standard temperature and pressure conditions. Aeration intensity was gradually increased, where each aeration step lasted 3 HRTs. The airflow increase in each step corresponded to one-third of the final aeration: 0.3, 0.6, and 1.0% when compared to the influent COD. Sludge extracted from the reactor at each aeration step (airflows of 0.3, 0.6, and 1.0%) was named S1, S2, and S3, respectively. Once the microaeration flux of 1% influent COD was reached, the MA-AnMBR was continuously operated under these conditions. The MA-AnMBR was considered to operate at stabilized performance after 100 days from the first aeration step. The period between the first added aeration and the stable conditions was called “adaptation,” and the period under stable microaeration conditions was denominated “MA-AnMBR state”. This period lasted 300 days, and the sludge extracted during this time was denominated S4. A schematic representation of the reactor periods is shown in Figure 2.

### 2.2 Analytical Methods

Total and volatile solids were measured according to standardized methods,^22^ and analysis was performed in triplicates. Sludge temperature, pH, and ORP were continuously measured with a Memosens CPS16D instrument (Endress+Hausser, Reinach, Switzerland). COD measurements were done using HACH Lange test kits LCK 314, 514, and 014 (HACH, Tiel, The Netherlands). Total phosphorus (TP), orthophosphate (PO_4_^3–^-P), total nitrogen (TN), ammonium-nitrogen (NH_4_^+^-N), and nitrate-nitrogen (NO_3_^–^-N) were measured with HACH Lange test kits (LCK 349, LCK238 LCK 303, and LCK 339). Samples were taken biweekly.

The composition of volatile fatty acids (VFA) in samples extracted from permeate and sludge was analyzed using Agilent tech 7890A gas chromatography (GC) (Agilent, Santa Clara, CA, U.S.A.) with helium as a carrier gas. The gas flow rate was 2.45 mL min^–1^, the pressure was 0.76 bar, and detector and injector temperatures were 225 and 240 °C, respectively. The samples were collected weekly and measured following the procedure described by García Rea.^23^

For biogas analysis, weekly gas samples were collected using 1.5 mL gas-lock syringes, after which they were immediately injected into a GC (Agilent, Santa Clara, CA, U.S.A) with a thermal conductivity detector (TCD). To analyze the composition of the gas samples, two separate columns, HP-PLOT U and a Molesieve GC column (Agilent 19095P-MS6, Santa Clara, CA, U.S.) of 60 m × 0.53 mm × 200 μm were used. Helium was used as a carrier gas at a flow rate of 10 mL min^–1^. The operational temperatures for the injector and detector were 40 and 200 °C, respectively.^24^

#### 2.2.1 Protein and Carbohydrate Degradation in Serum Bottles

Soluble and total protein concentrations in the reactor were measured via the modified Lowry method,^25^ while soluble and total carbohydrate concentrations were measured using the anthrone-sulfuric acid method by Dubois et al.,^26^ in serum bottles of 180 mL. A total of four aeration conditions were performed in triplicate during an incubation period equivalent to the AnMBR solids retention time (SRT), i.e., 28 days. Tests were performed to mimic the microaeration conditions of the laboratory-scale MA-AnMBR and to compare the effect of different aeration in the degradation of proteins and carbohydrates. Thus, four aeration conditions, called Ovalbumin A0-A3, were assessed. The supplied oxygen represented 0, 1, 2, and 5% of the substrate COD. As inoculum, 100 mL of sludge from the AnMBR period was used. The selected substrates were ovalbumin and micronutrients, mimicking the AnMBR synthetic influent concentrations. Ovalbumin was chosen as the main substrate due to its high ratio of added proteins to the influent. An inoculum over substrate ratio of 2 was considered, and the serum bottles were placed in a shaker at 110 rpm and 36 °C.^27^ Aeration was incorporated in pulses during the first six experimental days into the liquid phase. Produced biogas was removed twice per day during the first 10 days and afterward daily. Proteins and carbohydrates were measured at the beginning of the experiment and after 28 days of incubation. Finally, the measured concentrations of proteins and carbohydrates were converted to COD assuming the typical composition of protein (C_14_H_12_O_7_N_2_) and carbohydrate (C_10_H_18_O_9_), following Sophonsiri and Morgenroth.^28^

#### 2.2.2 Rheometry and Particle Size Distribution Analysis

A rotational rheometer model MCR 302 (Anton Paar GmbH, Graz, Austria) was used to measure the shear stress and shear rate. A smooth measuring cylinder model B-CC27 (0.026 m diameter) and a measuring cup model C-CC27 (0.030 m diameter) were used. A volume of 15 mL of sludge was used to perform the assay, which was done at 35 ± 0.2 °C. Since the sludge samples were stored in the fridge, a preshear stage was selected before starting the rheometric analysis. The methods followed were as described by Gonzalez et al.^29^

PSD was assessed with a Microtrac Bluewave diffraction analyzer (Malvern Instruments Ltd., UK), measuring particles between 0.01 and 2000 μm, via a light scattering technique. The results are shown as a volume-based PSD and are indicative of the presence of large-size particles. PSD was reported as percentiles D_10_, D_50_, and D_90_, where D_10_ represents the particle diameter of which only 10% of the particles are smaller than the given diameter.

#### 2.2.3 Specific Methanogenic Activity

SMA tests were performed to analyze the effects of different aeration rates on the SMA of the AnMBR sludge, under the different operating periods: AnMBR state, adaptation, and MA-AnMBR state. The tests were conducted for five triplicate sludge samples extracted from the laboratory-scale reactor. The first tests were conducted with the inoculum of AnMBR (S0). The second set of tests was performed using adapted sludge as an inoculum (S1–S3). Finally, the last inoculum used was the MA-AnMBR (S4).

An automated methane potential test system (AMPTS, Bioprocess Control, Sweden), at 37 °C, was used. Bottles of 250 mL with a liquid volume of 200 mL were used. Acetate, micro, and macronutrients were added as substrates following the method described by Spanjers and Vanrolleghem,^27^ and different aerations were incorporated in pulses. The aeration pulse was injected at the beginning of the SMA test in the liquid phase, and the bottles were sealed for 20 min while being constantly mixed at 80 rpm. Hereafter, the connections between the bottles and AMPTS were opened. Three aerations were selected to evaluate the SMA of the sludge and represented a ratio of oxygen over substrate COD of 3, 8, and 13%. These aerations were selected first to mimic the conditions of the lab-scale MA-AnMBR (3%) and to evaluate the inhibition of methane production due to different oxygen contents. Since the tests were performed in serum bottles of 250 mL, the volume error of injecting aerations below 3% of the substrate COD was considered inappropriate, and therefore, the minimum given aeration was set at 3%. All aeration conditions were compared to a positive control, where no aeration was added. The calculated amount of injected air was based on an oxygen content in the air of 21% and an oxygen density of 1.43g L^–1^ at 20 °C.

#### 2.2.4 Superoxide Dismutase Activity Analysis

SOD activity of the inoculum sludge acquired from a UASB at NIOO-KNAW (Wageningen, Netherlands) and the MA-AnMBR sludge was measured using a colorimetric method by Invitrogen (EIASOD, Thermo Fisher Scientific, Waltham, MA, U.S.A). Sample preparation was performed in triplicate and following the kit guidelines. One SOD unit is defined as the amount of enzyme causing inhibition of 50% in the reduction of 1.5 mM Nitro blue tetrazolium, in the presence of riboflavin at 25 °C and pH 7.8. SOD values were obtained in Units·mL^–1^ but final SOD activity was expressed in SOD Units mgProtein^–1^ as per Kato et al.^30^

### 2.3 Chemical Speciation

PhreeqC software was used to model the effect of microaeration on biogas composition and phosphorus speciation.^31^ PhreeqC enables the calculation of saturation indexes and distribution of aqueous species (among others) based on detailed influent characteristics and composition. The developed code was applied to four different scenarios, two of the AnMBR, and two of the MA-AnMBR states. The input data were derived from the synthetic influent characteristics and the laboratory-scale reactor characteristics (reactor and headspace volumes of 6.5 and 1.5 L, respectively). The PhreeqC code is described in the Supporting Information, Supporting Information C. Ammonium concentration in the liquid phase was taken from the reactor analytical measurements, being 583 and 740 mg L^–1^ for the AnMBR and the MA-AnMBR stable periods, respectively. Two initial biogas conditions were selected for each reactor period. Both conditions only include carbon dioxide and methane in ratios of 50:50 and 20:80. The final analyzed results were considered at pH values similar to the laboratory-scale experiments, i.e., 7.42 and 7.65 for the AnMBR state and MA-AnMBR state, respectively.

## 3 Results and Discussion

### 3.1 Reactor Performance

Variations in reactor pH, ORP, and maximum biogas production under the three studied periods (AnMBR, Adaptation, and MA-AnMBR states) are shown in Table 2. The permeate of the laboratory-scale AnMBR reached COD values below 100 mg L^–1^ on average, corresponding to a removal efficiency exceeding 98% (Table 2). Single-factor ANOVA was used to assess the statistical difference between the reactor parameters at different periods. Thus, a statistically relevant increase of 0.2% in COD removal was observed during the MA-AnMBR state when compared to the AnMBR conditions (*p*-value < 0.01). The COD permeate concentrations of the AnMBR and the MA-AnMBR were 90.6 ± 4.4 and 74.6 ± 19.0 mgCOD L^–1^. A summary of the permeate characteristics for the AnMBR and MA-AnMBR are further described in Supporting Information D. An added amount of oxygen of 1.0% of the influent COD load corresponds to a potential aerobic degradation of 112 mgCOD d^–1^, based on kinetics and stoichiometric constants given by Ekama and Wentzel.^32^ Since the difference between permeate COD of both AnMBR and MA-AnMBR amounted to about 40 mgCOD d^–1^, the increased COD removal might be attributed to microaerobic conversion using the added oxygen as an electron acceptor.

**Table 2:** Summary of the Reactor Performance during the Different Operational Periods^a^

		unit	AnMBR	adaptation	MA-AnMBR
operation time		days	180	100	300
**sludge pH**			**7.42 ± 0.02**	**7.52 ± 0.14**	**7.65 ± 0.13**
sludge oxidation–reduction potential (ORP)		mV	–516 ± 44	–533 ± 16	–533 ± 42
maximum biogas production		L d^–1^	3.6	3.7	3.0
**average biogas production**		**L d**^**–1**^	**2.5 ± 0.8**	**2.1 ± 0.8**	**1.8 ± 0.5**
**chemical oxygen demand (COD) removal efficiency**		**%**	**98.2 ± 0.1**	**98.3 ± 0.1**	**98.5 ± 0.4**
**ortho-phosphate removal**		**%**	**16.8 ± 5.4**	**24.3 ± 1.3**	**48.3 ± 18.8**
sulfate removal		%	88.4 ± 0.6	89.3 ± 0.3	89 ± 4.7
**ammonium concentration increase factor**			**2.3**	**2.6**	**3.0**
VFA content in the mixed liquor		mgCOD L	5.6 ± 1.9	15.0 ± 17.9	6.6 ± 1.1
methane concentration in biogas		%	82 ± 2	84 ± 3	84 ± 6
**sludge total solids**		**g L**^**–1**^	**4.8 ± 1.0**	**5.9 ± 0.3**	**5.8 ± 0.9**
sludge volatile solids		g L^–1^	2.7 ± 0.6	3.6 ±0.3	2.9 ± 0.6
**ash content**		**g L**^**–1**^	**2.1 ± 1.2**	**2.3 ± 0.4**	**2.9 ± 1.1**
**particle size distribution**^b^	**D**_**90**_	**μm**	**10.6 ± 0.7**	**13.0 ± 1.4**	**19.5 ± 0.6**
	**D**_**50**_	**μm**	**4.4 ± 0.3**	**5.2 ± 0.8**	**6.7 ± 0.2**
	**D**_**10**_	**μm**	**2.7 ± 0.2**	**3.2 ± 0.4**	**4.3 ± 0.1**

a Values correspond to averages and standard deviation of samples (in triplicates) taken bi-weekly during each period. Information regarding the studied periods is shown in Figure 2. For particle size distribution, values of D_90_, D_50_, and D_10_ represent the particle diameters at which 90, 50, and 10% of the total number of particles are smaller than the given diameter respectively. Values shown in bold correspond to those which had statistically important changes between the different reactor periods.

b Values based on total particle numbers.

Single-factor ANOVA test showed no statistical difference between the ORP of all reactor periods. All three reactor periods showed values below −500 mV, and therefore, prevailing conditions could be considered fully anaerobic.^33^ These results align with the research conducted by Lim and Wang,^17^ who observed negligible ORP variations when microaeration corresponding to 1.0% of soluble influent COD load was added as a pretreatment of anaerobic digestion. In our present research, anaerobic conditions were maintained during the adaptation period, and the reactor ORP remained around −533 mV, showing that the oxygen introduced to the reactor was rapidly consumed and undetectable in the higher part of the liquid phase, where the ORP probe was located. However, the adaptation period was characterized by a slight increase in VFA concentrations during the first month of microaeration. Iso Caproic acid (I C6) and Caproic Acid (C6) increased to a maximum value of 50 mg L^–1^ (after 1 week of starting aeration) and decreased to negligible values after this first adaptation. Under the MA-AnMBR state, VFA concentrations were negligible.

While no statistical changes were observed in the reactor ORP, biogas quantity decreased during the MA-AnMBR period. Average biogas production decreased by 25%, from 2.5 to 1.8 L d^–1^ in the AnMBR and MA-AnMBR periods, respectively. A high relative standard deviation of biogas production was observed during operation of the reactor (30%). This was mainly due to tube obstructions (primarily on the feed line) and reactor headspace variations due to daily operation and maintenance.

No statistical difference was observed in the biogas quality (p-value of 0.13), which showed high methane concentrations reaching 82 ± 2 to 84 ± 6% for the AnMBR and MA-AnMBR states, respectively. Similar observations were made by Ferrari et al.^34^ who found methane concentration in the biogas between 85 and 95% while operating a laboratory-scale AnMBR treating concentrated synthetic municipal sewage in the temperature range 17–34 °C and HRT from 1 to 1.5 days. Methane concentrations reaching 70–80% are commonly found at full-scale anaerobic reactors treating dilute municipal sewage at relatively low HRTs, which can be attributed to the relatively high CO_2_ solubility in the effluent.^35^ The resulting CO_2_ concentration in the biogas of these reactors is only 5–10%, while the remainder consists of atmospheric N_2_ gas that was dissolved in the influent. The observed high methane concentrations in our present study and that of Ferrari^38^ might be attributed to the presence of urea, which was used as the main nitrogen source in the synthetic influent. It should be noted that each mmol of urea is hydrolyzed in two mmol of NH_4_^+^, which increases the alkalinity, produces one mmol of HCO_3_^–^, and chemically binds two mmol of CO_2_ as bicarbonate to the liquid. The proteins present in the influent will generate NH_4_^+^, which concomitantly binds CO_2_.

The main protein sources of the influent were ovalbumin (200 mg L^–1^), milk powder (600 mg L^–1^), and yeast extract (510 mg L^–1^). The protein percentage of milk powder and yeast extract is around 25% w/w.^36,37^ Food proteins contain 16% nitrogen (by weight);^38^ therefore, the NH_4_^+^ generated by the influent proteins represents less than 15% of the total ammonium produced by urea, thus having a minor impact on the binding of CO_2_. Research showed that under a high acid neutralization capacity (ANC) to total inorganic produced carbon (TIC) ratio, a decrease in carbon dioxide content in the biogas can be expected.^39^ The observed methane concentrations in the biogas of the AnMBR and MA-AnMBR are therefore in line with the literature.

An increase in carbon dioxide in the biogas of less than 50 mL d^–1^ can be expected under aerobic respiration due to the added oxygen in the AnMBR (representing around 1% of the influent COD). To better understand the biogas composition, the AnMBR and MA-AnMBR conditions were modeled using the ammonium database of the PhreeqC software. Results of the model showed concentrations of CH_4_ and CO_2_ of 87.8 and 6.7%, respectively, for the AnMBR, and 90.0 and 4.5% for the MA-AnMBR state. Relatively low discrepancies between the model outputs and observed biogas composition of 5.8 and 6% for the AnMBR and MA-AnMBR, respectively, were observed. Further discussions on the PhreeqC model results and biogas composition dependency on feed characteristics can be found in the Supporting Information, Supporting Information E.

Finally, the COD mass balance was calculated for the AnMBR and MA-AnMBR states. For the biogas COD, the maximum daily biogas production was considered instead of the average values due to the high standard deviation (due to operational and maintenance issues). Influent, permeate, and sludge flows were considered as defined by the operation conditions specified in Table 1. The difference in biogas production between the AnMBR and MA-AnMBR corresponded to a COD load of around 1300 mgCOD d^–1^, while theoretical calculations of aerobic degradation due to the supplied oxygen load corresponded to a potential degradation of 112 mgCOD d^–1^. Thus, while aerobic degradation could have contributed to the observed decrease in biogas production (and methane content), the changes in the biogas quantity cannot be attributed to aerobic degradation of the influent COD alone. Results showed an off-balance of 5% for AnMBR and 9% for MA-AnMBR when compared to the influent COD load. Biogas COD corresponded to 93 and 79% of the influent COD at the AnMBR and MA-AnMBR states, respectively, while the permeate and sludge COD loads did not vary significantly between the reactor states. The COD balance for each reactor period is presented in Supporting Information F.

### 3.2 Effects of Microaeration on Physical Sludge Characteristics

Solids concentration, particle size, and viscosity varied for each of the reactor periods. While the sludge total solids concentration increased with microaeration, from 4.6 ± 0.3 to 5.8 ± 0.5 g L^–1^, no significant change was observed in the volatile solids. Changes in the TSS content are in line with the increased precipitation of phosphate compounds (like hydroxyapatite). The supplied oxygen would maximally increase 0.04 gVSS L^–1^ of aerobic biomass, assuming a yield of 0.5 gVSS gCOD^–1^,^6^ which is considered negligible for the prevailing AnMBR and MA-AnMBR TSS concentrations, 4.8 and 5.8 gTSS L^–1^ respectively.

Sludge viscosity and PSD changed from AnMBR to the MA-AnMBR period. Based on the rheometer results, MA-AnMBR sludge viscosity decreased when compared to the AnMBR. Under shear rates of 1.0 and 100.0 s^–1^, MA-AnMBR showed shear stress values of 0.001 and 0.098 Pa, respectively, while for the AnMBR sludge, these values were 0.031 and 0.205. The shear stress against the shear rate for the different sludges is shown in Supporting Information G. Furthermore, PSD of the MA-AnMBR sludge showed a statistical increase in D_10_, D_50_, and D_90_ compared to the AnMBR sludge (p-values below 0.05). The highest difference was observed for the D_90_ particles, where the average particle diameter from the MA-AnMBR sludge, i.e., 19.5 μm, was almost 90% larger than the ones from the AnMBR sludge, i.e., 10.6 μm (Table 2). A lower apparent viscosity can be potentially linked to better mixing and higher biogas production rates.^40^

### 3.3 Effects of Microaeration in Substrate Degradation and Sludge-Specific Methanogenic Activity

#### 3.3.1 Substrate Degradation Potential with Microaeration

**Figure 3:**
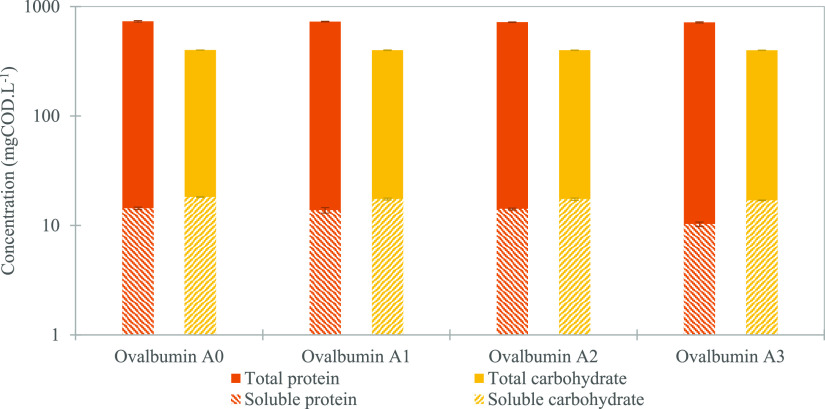
Concentrations of total and soluble proteins and carbohydrates. The substrate used for the batch experiments was ovalbumin, macronutrients, and micronutrients, in a composition similar to that selected for the reactor feed. The inoculum used was the AnMBR state sludge. The batch tests were conducted for 28 days, and aeration took place on the first 6 days. Values displayed are the mean over the triplicate samples followed by the standard deviation.

Following the observed changes in ammonium and phosphate concentrations between the AnMBR and MA-AnMBR operational periods, protein degradation was assessed. One of the main protein sources in the synthetic influent was ovalbumin; the degradation of which was tested under different aeration conditions in batch tests. Results showed that protein and carbohydrate concentrations in sludge decreased when aeration increased (Figure 3). When no aeration was applied (further referred to as Ovalbumin A0), total proteins and carbohydrate concentrations were 732 ± 10 and 400 ± 1 mgCOD L^–1,^ respectively. While degradation of total proteins and carbohydrates seemed to increase with aeration, the change was statistically insignificant (*p*-value above 0.05). Nevertheless, the difference was notable in the soluble fraction. Soluble protein concentration was 10.3 mgCOD L^–1^ when the supplied oxygen was 5% of the substrate COD load (further referred to as Ovalbumin A3), and 14.3 mgCOD L^–1^ for Ovalbumin A0, showing a significant 28% reduction (*p*-value < 0.001). Moreover, soluble carbohydrates concentration decreased from 18.2 to 17.0 mgCOD L^–1^ in A0 and A3 respectively, (*p*-value < 0.05). Thus, the added aeration contributed to an increased degradation of soluble protein and carbohydrates.

#### 3.3.2 Sludge-Specific Methanogenic Activity under Different Aeration Conditions

**Figure 4:**
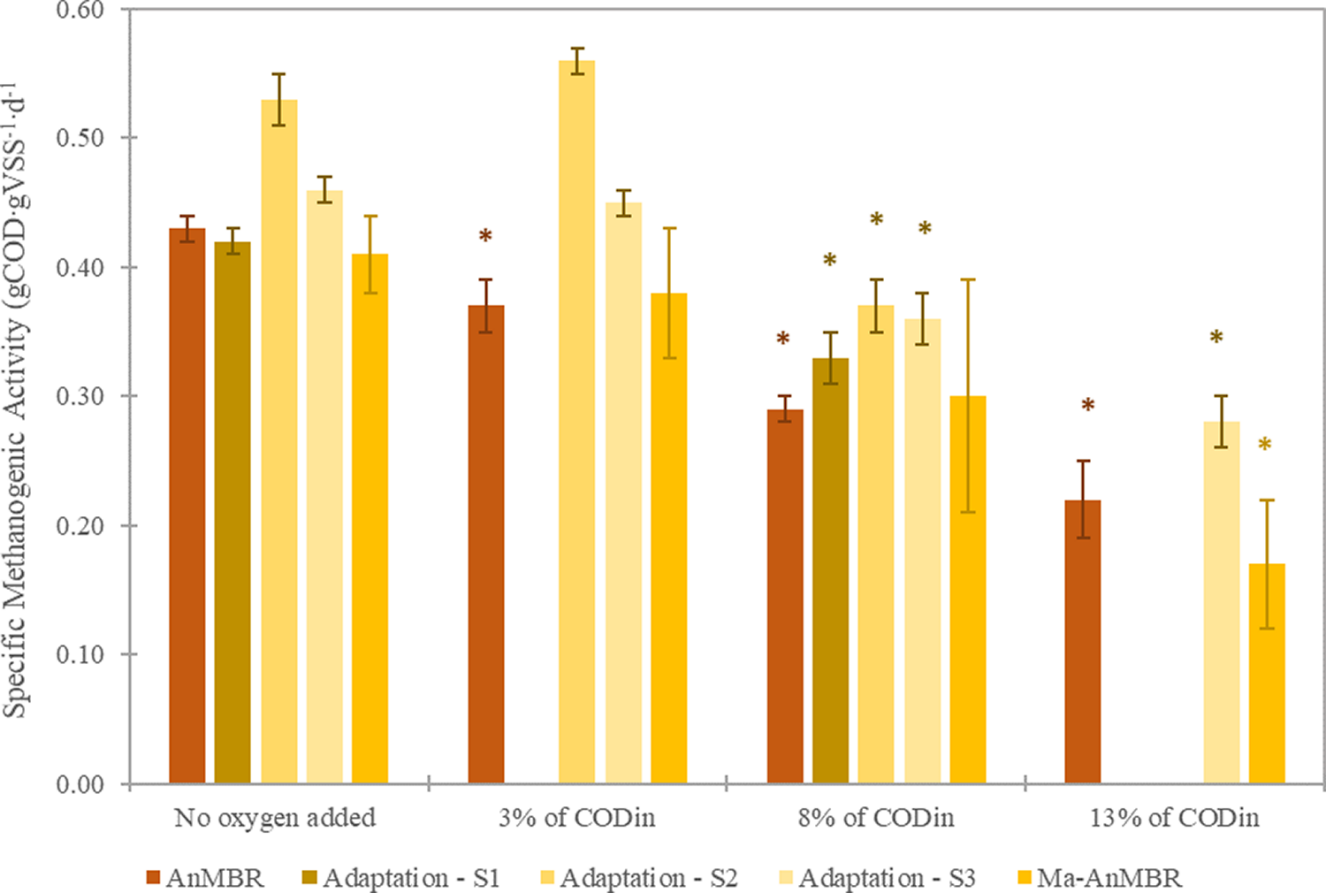
SMA in gCOD gVSS^–1^ d^–1^ of sludge inocula under different aerations. The oxygen supplied to each SMA bottle was calculated based on the oxygen overload of substrate COD (COD_in_) ratio, at 20 °C, and considering an air composition of 21% oxygen and 79% nitrogen. Blank results under the different aerations indicate no measurements. Columns with an * show that the difference between the SMA of the sample was significant when compared to the no added oxygen conditions (*p* values < 0.05).

The SMA of the MA-AnMBR sludge was measured under different aeration conditions, using biomass from the laboratory-scale reactor as inoculum that was harvested from the AnMBR stage (S0), during adaptation (S1–S3) and after full adaptation to microaeration (S4). The SMA results can be seen in Figure 4. The aeration during the SMA test corresponded to ratios of oxygen over substrate COD loads of 3, 8, and 13%. For any given inoculum, an increase in the level of aeration resulted in a decreased SMA.

Significant SMA decreases (with *p*-values below 0.01) of 14, 33, and 48% for the AnMBR state inoculum were observed with increasing aerations corresponding to 3, 8, and 13% of substrate COD load, respectively. The tests performed with inoculums from the Adaption period showed a statistically significant decrease in SMA for all three stages (S1, S2, and S3) of 20 and 35% when added oxygen corresponded to 8 and 13% of substrate COD, respectively (*p*-values below 0.01). No statistical variation was observed for the aeration corresponding to 3% substrate COD (*p*-values above 0.4). SMA results showed a tendency toward biomass adaptation to small amounts of added oxygen. A negligible impact on SMA was also observed for the MA-AnMBR state sludge for the lowest aeration (*p*-value of 0.6). Furthermore, this inoculum also had an insignificant reduction in SMA when the added oxygen was 8% of the substrate COD. Nevertheless, the absence of significant differences could be linked to the high standard deviations. Relative standard deviations above 20% were observed in all SMA tests conducted for the MA-AnMBR state inoculum subjected to an oxygen increase of 8% of substrate COD, which was performed five times and in triplicates. Finally, IC_50_, an SMA decrease above 50% (*p*-value of 0.04), was observed for the MA-AnMBR state inoculum exposed to oxygen that corresponded to 13% of the substrate COD load. All p-values are given in the Supporting Information, Supporting Information H.

Even though SMA deterioration was observed for added oxygen contents of 8 and 13%, no variation in the lag-phases in the methane production of the different inoculums was observed. Furthermore, the negligible change in SMA of the MA-AnMBR sludge subjected to 3% oxygen over substrate COD load suggested that the acetotrophic methanogenic biomass can tolerate small amounts of added oxygen, apparently creating resistance toward it.

### 3.4 Nutrient Removal in the MA-AnMBR vs AnMBR

Although the difference in total protein concentration between MA-AnMBR and AnMBR was statistically insignificant, the change in soluble proteins showed an increase in protein degradation when oxygen was added to the reactor (see Figure 3). Soluble protein concentration in the microaerated sludge was 30–35% lower than the one in the AnMBR sludge. It is hypothesized that the 30% reduction in soluble protein concentration is directly linked to an increase in the hydrolytic capacity of the biomass and the decrease in the permeate COD from 90.6 to 74.6 mgCOD L^–1^. This hypothesis was confirmed by the fact that the ammonium concentration during the MA-AnMBR period was 1.3 times higher than that during the AnMBR period. Hydrolysis is commonly considered the rate-limiting step when treating wastewater with high concentrations of particulate matter.^41^ Since hydrolysis occurs under a wide range of redox conditions, the observed increased protein hydrolysis, suggests that the addition of oxygen may enhance the hydrolysis rate of protein and thus the overall digestion performance.^42,43^

The results of PhreeqC modeling showed that the reactor broth was supersaturated for, among others, hydroxyapatite, Ca_5_(PO_4_)_3_OH, under both AnMBR and MA-AnMBR periods. Furthermore, the saturation indexes (SI) increased by 12% to 6.4, under the MA-AnMBR operational conditions compared to the AnMBR state, with pH values of 7.65 and 7.43, respectively. Aside from hydroxyapatite, aragonite, and calcite (carbonate minerals) saturation indexes increased from the AnMBR to the MA-AnMBR periods, from 0.4 and 0.5 to 0.5 and 0.7, respectively. This increase in carbonate minerals can be further linked to a decrease in the partial pressure of CO_2_ in the biogas. Since under all conditions, ORP levels showed values below −250 mV, it can be assumed that anaerobic conditions were maintained in the laboratory-scale reactor. Hydroxyapatite SI indicated precipitation of the mineral under both AnMBR and MA-AnMBR states. The precipitation of hydroxyapatite can be further linked to an increase in the concentration of calcium and phosphate in the sludge of the laboratory-scale reactor. When compared to the AnMBR, the phosphate concentration in MA-AnMBR permeate reduced to half, from 55.1 to 27.6 mgPO_4_^3–^-P L^–1^.

### 3.5 Increase in Superoxide Dismutase Activity

Microbial SOD activity of the MA-AnMBR sludge increased by a factor of 3 compared to that of the AnMBR inoculum sludge, i.e., 4.3 ± 0.4 and 1.4 ± 0.1 U mgProtein^–1^, respectively. An increase in SOD activity of the MA-AnMBR sample is linked to a higher amount of antioxidant enzymes that protect against oxidant stress situations.^44^ Enzymatic production requires an extensive energy investment in enzyme synthesis and excretion, consuming up to 5% of bacterial productivity.^45,46^ Thus, an increase in the SOD activity can be linked to an additional need for organic matter from the microorganisms to produce the enzyme, resulting in a lower sludge yield.

The surge in SOD activity is associated with a higher tolerance to oxygen since it is likely related to the neutralization of superoxide anion radicals and a localized decrease in redox potential.^47^ Since no changes in the reactor ORP were observed, a rise in the enzyme activity could be responsible for regulating the oxygen tolerance of the biomass at a localized level. Results of the SOD activity can also be linked to MA-AnMBR sludge SMA. The negligible decrease in SMA of the MA-AnMBR subjected to an oxygen load equivalent to 3% of the substrate COD load indicated an increase in oxygen tolerance of the MA-AnMBR methanogenic biomass, and it could therefore be related to the rise in SOD activity. Even though SMA changes were negligible, biogas production in the lab-scale reactor decreased by 25% during MA-AnMBR versus AnMBR operation. This decrease potentially might be attributed to an increased anabolism to produce the SOD enzyme as well as microbial community shifts, but further studies are necessary to verify this.

For further information about the effect of air supplied into the AnMBR on the microbial community of the MA-AnMBR, see Supporting Information G.

## 4 Conclusions

A study was conducted on the effects of microaeration on a laboratory-scale anaerobic membrane bioreactor (MA-AnMBR), where the given oxygen load mimics the conditions of an anaerobic digester–dissolved air flotation system (AD-DAF). The main conclusions of the research are as follows:

The microaeration addition (55 mLO_2_ d^–1^) showed negligible effects on the operation of the MA-AnMBR, and performance remained stable. Permeate COD decreased from 90.6 ± 4.4 during AnMBR to 74.6 ± 19.0 mgCOD L^–1^ during MA-AnMBR operation. Furthermore, the produced biogas quantity decreased by 27%, which could not be solely attributed to aerobic conversions.
Ammonium concentration in the permeate increased from 547 to 740 mgNH_4_^+^-N L^–1^, following AnMBR to MA-AnMBR, respectively. This suggests a higher hydrolytic capacity of protein in the latter. The increased ammonium concentration led to a further increase in buffer capacity and pH, subsequently decreasing biogas CO_2_ content further.
When compared to the AnMBR, orthophosphate concentration in MA-AnMBR permeate was halved, from 55.1 to 27.6 mgPO_4_^3–^-P L^–1^. The measured change in pH from 7.42 to 7.65 resulted in increased hydroxyapatite precipitation in the MA-AnMBR period and a decrease in the permeate orthophosphate concentration.
MA-AnMBR sludge adapted to given oxygen exposure. Microbial adaptation was revealed by the increase in superoxide dismutase (SOD) enzyme activity, which tripled from AnMBR to MA-AnMBR operation (1.4 ± 0.1 and 4.3 ± 0.4 U mgProtein^–1^ respectively). Furthermore, the specific methanogenic activity (SMA) of the MA-AnMBR sludge was not affected despite an oxygen exposure of 3% of the substrate COD load.

Oxygen inhibition was considered a major concern for the feasibility of employing the DAF as an alternative solid retention mechanism in anaerobic bioreactors. All obtained results in AnMBR showed that the provided oxygen loads, mimicking the coupling of an anaerobic digester with a DAF (AD-DAF), had negligible effects on the performance of the anaerobic conversion process. Owing to the promising hydraulic characteristics, the potential of AD-DAF deserves further exploration in a representatively sized system.

## Supporting Information Available

Additional experimental details; materials and methods; additional information on experimental results; effluent and sludge characteristics; modeling of the reactor performance using PhreeqC; and microbial community (PDF)
