# Supplementary material for: Effects of Low Oxygen Dosages on an Anaerobic Membrane Bioreactor, Simulating the Oxygen Load in an Anaerobic Digester-Dissolved Air Flotation (AD-DAF) System
Source: ACS ES T Water. 2023 Nov 11;3(12):4133–42. doi: 10.1021/acsestwater.3c00544 (PMC10714394; doi:10.1021/acsestwater.3c00544)
Supplement: Supplementary file 1 — ew3c00544_si_001.pdf [file ew3c00544_si_001.pdf]

SUPPLEMENTARY INFORMATION

Effects of low oxygen dosages on an anaerobic membrane bioreactor, simulating the oxygen load in an anaerobic digester-dissolved air flotation (AD-DAF) system.

Antonella L. Piaggio[1], K.B. Sasidhar[1,2], Pravin Khande[1,3], Malini Balakrishnan[4], Jules B. van Lier[1], Merle K. de Kreuk[1], Ralph E.F. Lindeboom[1]*

[1]Delft University of Technology, Faculty of Civil Engineering and Geosciences, Section Sanitary Engineering, Department of Water Management, Stevinweg 1, 2628 CN Delft, The Netherlands.

[2]School of Civil Engineering, Vellore Institute of Technology, Vellore, 632 014, Chennai, India.

[3]NX Filtration, Nanotechnology research, Josink Esweg 44, 7545 PN Enschede, The Netherlands

[4]The Energy and Resource Institute (TERI), Darbari Seth Block, IHC Complex, Lodhi Road, New Delhi-110003, India.

SUPPORTING INFORMATION A

Calculations of the added oxygen to the AnMBR to simulate the oxygen dosage from an AD-DAF system.

Based on previous studies and literature regarding DAF design [1]. The lab-scale DAF and AD design parameters were stablished and are shown in **Table A.1.** and **Table A.2.**

Table A.1. *Lab-scale DAF design parameters*

	Unit	Value
DAF system shape	-	cylinder
DAF hydraulic retentnion time	d	0.04
DAF height over diameter ratio	-	2

Recycle ratio*	%	50
White water pressure	bar	5
White water temperature	°C	37.0
Air density	$g{\cdot}L^{-1}$	1.2
Oxygen density	$g{\cdot}L^{-1}$	1.31

*Recycle ratio entitles the ratio between the influent flow (Q_{in}), and the effluent flow, defined as the sum of the influent flow plus the white water (recycle) flow (Q_{in}+Q_{rec})

Table A.2. *AD design parameter*

	Unit	Value
Influent COD	$mg{\cdot}L^{-1}$	5000
Hydraulic retention time	d	2.6
Working volume	L	6.5
Solids retention time	d	27

The chosen AD conditions mimic the operational conditions of the AnMBR. The effluent flow of the AD (which is similar to the permeate flow of the AnMBR), was set as influent flow to the DAF system (Q_p). Thus, based on the design parameters shown in Tables A.1 and A.2, the lab-scale DAF had a volume of 0.19 L, a height of around 10 cm, and a diameter od around 5 cm. The final DAF influent entitles the before mentioned effluent flow plus the recirculation flow, as shown in **Figure A.**

Figure A. *Flow balance of the AD-DAF system*

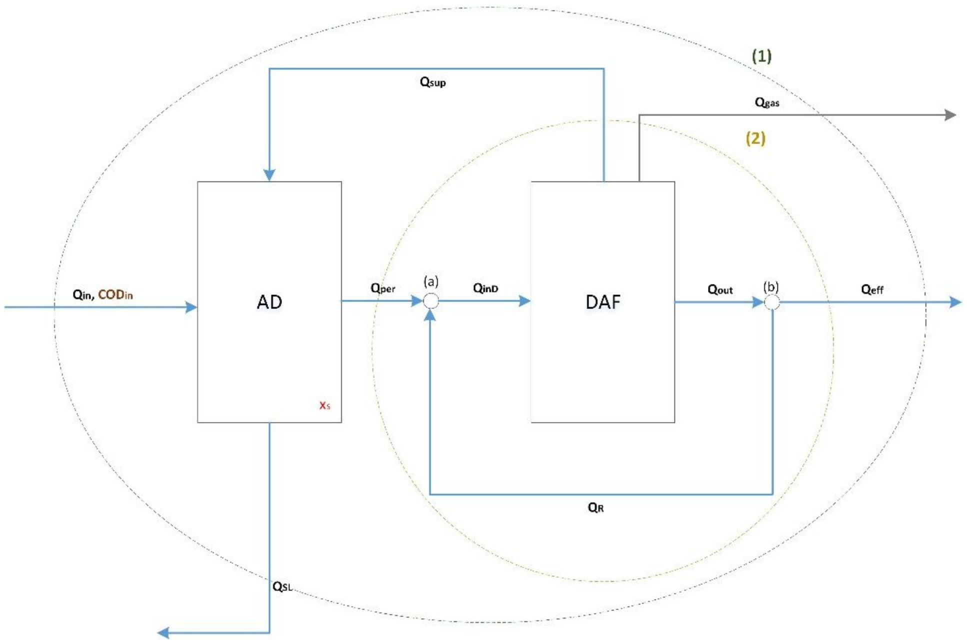

To calculate the oxygen load in the DAF system the following assumptions were made:

- No oxygen escapes the system (Q_{gas}→0). This would be the worse case scenario in which the anaerobic biomass in the AD would be subjected to the highest amount of oxygen.
- Air is assumed to be composed of 21% oxygen and 79% nitrogen.
- Dissolution of air at the gicen pressure (or air transfer efficiency) is assumed to be at 70% in accordance to Wang, et al. [2].
- Dissolved oxygen and nitrogen were calculated based on Henry's constants for oxygen and nitrogen (1.2×10^{-3} and 6.5×10^{-4}, respectively) and correcting for the desire temperature [3].

Considering the above mentioned assumptions, **Table A.3** shows the oxygen load calculations and comparison to the AD COD inflow.

Table A.3. Oxygen load calculations in the lab-scale DAF

	Unit	Value
$Q_{permeate}$ - Q_p	$L\cdot d^{-1}$	4.52

Q_{reycle} - Q_r	$L \cdot d^{-1}$	4.52
O_2 concentration in $Q_{recycle}$	$g \cdot L^{-1}$	0.031
O_2 load in $Q_{recycle}$	$g \cdot d^{-1}$	0.139
$Q_{supernatant}$	$L \cdot d^{-1}$	2.26
Q_{out}	$L \cdot d^{-1}$	6.78
$Q_{effluent}$	$L \cdot d^{-1}$	2.26
Q_{inD}	$L \cdot d^{-1}$	9.04
O_2 concentration $Q_{in\ (DAF)}$	$g \cdot L^{-1}$	0.015
O_2 % base on CODin of AD	%	1.1%

SUPPORTING INFORMATION B

***Table B.1**. Micro-aerated AnMBR feed recipe.*

Feed composition	Unit	Value	Micronutrients Solution	Unit	Value
Urea	$g \cdot L^{-1}$	1.0	$FeCl_3.6H_2O$	$mg \cdot L^{-1}$	1000.0
Ammonium chloride	$g \cdot L^{-1}$	0.8	$CoCl_2.6H_2O$	$mg \cdot L^{-1}$	1000.0
Sodium acetate trihydrate	$g \cdot L^{-1}$	2.6	$MnCl_2.4H_2O$	$mg \cdot L^{-1}$	250.0
Ovalbumin	$g \cdot L^{-1}$	0.2	$CuCl_2.2H_2O$	$mg \cdot L^{-1}$	15.0
Magnesium sulphate heptahydrate	$g \cdot L^{-1}$	0.1	$ZnCl_2$	$mg \cdot L^{-1}$	25.0
Potassium phosphate monobasic	$g \cdot L^{-1}$	0.2	H_3BO_3	$mg \cdot L^{-1}$	25.0
Calcium chloride dihydrate	$g \cdot L^{-1}$	0.1	$(NH_4)_6Mo_7O_{24}.4H_2O$	$mg \cdot L^{-1}$	45.0
Cellulose	$g \cdot L^{-1}$	1.5	$Na_2SeO_3.H_2O$	$mg \cdot L^{-1}$	50.0
Milk powder	$g \cdot L^{-1}$	0.6	$NiCl_2.6H_2O$	$mg \cdot L^{-1}$	25.0
Yeast extract	$g \cdot L^{-1}$	0.5	EDTA	$mg \cdot L^{-1}$	500.0
Sunflower oil	$drops \cdot L^{-1}$	2.0	HCI 36%	$mg \cdot L^{-1}$	0.5
Humic and Fulvic acid	$drops \cdot L^{-1}$	2.0	Resazurin sodium salt	$mg \cdot L^{-1}$	250.0
Micronutrients solution	$g \cdot L^{-1}$	10.6	Yeast extract	$mg \cdot L^{-1}$	1000.0

***Table B.2**. Micro-Aerated AnMBR feed composition*

	Unit	Value
Chemical Oxygen Demand (COD)	$mg \cdot L^{-1}$	5200 ± 600
Ammonium (NH_4^+)	$mgN \cdot L^{-1}$	249± 54
Nitrate (NO_3^-)	$mgN \cdot L^{-1}$	1.3 ± 0.2
Phosphate (PO_4^{3-})	$mgP \cdot L^{-1}$	60 ± 9
Sulphate (SO_4^{2-})	$mgS \cdot L^{-1}$	235 ± 46
Total Suspended Solids (TSS)	$mg \cdot L^{-1}$	3073 ± 451
Volatile Suspended Solids (VSS)	$mg \cdot L^{-1}$	2938 ± 436

SUPPORTING INFORMATION C

***Code B.1**. PhreeqC model for the AnMBR state*

```
GAS_PHASE 1
Fixed_Pressure
    -fixed_pressure            # 0.33 L (default: 1.0 L)
    -pressure 1.05
    CO2(g) -0.282017616    # 50% (1.05bar)
    H2S(g) -0.0      # not deteectable in GC(1.05bar)
    Amm(g) -0.00   # 0% (1.05bar)
    CH4(g) -0.282017616    # 50% (1.05bar)
SOLUTION 1
pH 4.0
Temp 36.5
units mg/L
C(4) 2350
C(-4) 932
Ca 27.3
Co 0.0
S(-2) 13.01
Fe(+2) 0.86
Mn(+2) 0.73
Amm 583   # NH4+
Pb 0.001
Cu 0.06
Cr 0.0
Cd 0.0
Zn 0.12
Cl 587.35
Mg 9.87
Si 0.00
B 0.05
Ba 0.0
Al 0.01
Na 439.84
Ni 0.06
Sr 0.01
Ti 0.01
K 57.47
P 139.59 as PO4
N 714.71
SELECTED_OUTPUT
       -file selectedoutput.sel
       -temperature
INCREMENTAL_REACTIONS True  # you can also choose False, if you do not want cumulative additions)

REACTION

NaOH 1.0; 1.0 moles in 1000 steps

USER_GRAPH 1
 -headings head CO2 HCO3- CO3-2 CaHCO3+ CO2(g)
 -chart_title "pH effect carbon speciation"
 -axis_titles "pH" "CO2 speciation (mol)" "Partial pressure (atm)"
-axis_scale x_axis      5 14 auto auto
    -axis_scale y_axis      0.0 0.07 auto auto
-axis_scale sy_axis     0.00 0.7 auto auto
    -initial_solutions      true
    -connect_simulations    true
    -plot_concentration_vs  x
  -start
```

```
10 graph_x -LA("H+")
20 graph_y  MOL("CO2") MOL("HCO3-") MOL("CO3-2") MOL("CaHCO3+")
30 graph_sy  PR_P("CO2(g)")

USER_GRAPH 2
 -headings head H2S HS- S-2 Fe(HS)2 H2S(g)
 -chart_title "pH effect sulphur speciation"
 -axis_titles "pH" "S-speciation (mol)"  "Partial Pressure (atm)"
-axis_scale x_axis      5 14 auto auto
    -axis_scale y_axis      0 0.0016 auto auto
-axis_scale sy_axis     0 0.011 auto auto
    -initial_solutions      true
    -connect_simulations    true
    -plot_concentration_vs  x
  -start
10 graph_x -LA("H+")
20 graph_y  MOL("H2S") MOL("HS-") MOL("S-2") MOL("Fe(HS)2")
30 graph_sy PR_P("H2S(g)")

USER_GRAPH 3
 -headings head NH3(l) NH4+ NH3(g)
 -chart_title "pH effect ammonia speciation "
 -axis_titles "pH" "NH4-speciation (mol)"  "Partial pressure (atm)"
-axis_scale x_axis      4 14 auto auto
    -axis_scale y_axis      0 0.05 auto auto
-axis_scale sy_axis     0 0.001 auto auto
    -initial_solutions      true
    -connect_simulations    true
    -plot_concentration_vs  x
  -start
10 graph_x -LA("H+")
20 graph_y  MOL("Amm") MOL("AmmH+")
30 graph_sy PR_P("Amm(g)")

USER_GRAPH 4
 -headings head SI("Calcite") SI("Aragonite") SI("FeS(ppt)")
 -chart_title ""
 -axis_titles "pH" "Saturation index"
-axis_scale x_axis      3 14 auto auto
    -axis_scale y_axis      -10 10 auto auto
-initial_solutions      true
    -connect_simulations    true
    -plot_concentration_vs  x
  -start
10 graph_x -LA("H+")
20 graph_y  SI("Calcite") SI("Aragonite") SI("FeS(ppt)")

END
```

Code B.2. *PhreeqC model for the Ma-AnMBR state*

```
Fixed_Pressure
    -fixed_pressure            # 0.33 L (default: 1.0 L)
    -pressure 1.05
    CO2(g) -0.282017616    # 50% (1.05bar)
    H2S(g) -0.0      # not deteectable in GC(1.05bar)
    Amm(g) -0.00   # 0% (1.05bar)
    CH4(g) -0.282017616    # 50% (1.05bar)
SOLUTION 1
pH 4.0
Temp 36.5
units mg/L
C(4) 2350
```

```
C(-4) 932
Ca 27.3
Co 0.0
S(-2) 13.01
Fe(+2) 0.86
Mn(+2) 0.73
Amm 714   # NH4+
Pb 0.001
Cu 0.06
Cr 0.0
Cd 0.0
Zn 0.12
Cl 587.35
Mg 9.87
Si 0.00
B 0.05
Ba 0.0
Al 0.01
Na 439.84
Ni 0.06
Sr 0.01
Ti 0.01
K 57.47
P 139.59 as PO4
N 714.71
SELECTED_OUTPUT
       -file selectedoutput.sel
       -temperature
INCREMENTAL_REACTIONS True  # you can also choose False, if you do not want cumulative
additions)

REACTION

NaOH 1.0; 1.0 moles in 1000 steps

USER_GRAPH 1
 -headings head CO2 HCO3- CO3-2 CaHCO3+ CO2(g)
 -chart_title "pH effect carbon speciation"
 -axis_titles "pH" "CO2 speciation (mol)" "Partial pressure (atm)"
-axis_scale x_axis      5 14 auto auto
    -axis_scale y_axis      0.0 0.07 auto auto
-axis_scale sy_axis     0.00 0.7 auto auto
    -initial_solutions      true
    -connect_simulations    true
    -plot_concentration_vs  x
  -start
10 graph_x -LA("H+")
20 graph_y  MOL("CO2") MOL("HCO3-") MOL("CO3-2") MOL("CaHCO3+")
30 graph_sy  PR_P("CO2(g)")

USER_GRAPH 2
 -headings head H2S HS- S-2 Fe(HS)2 H2S(g)
 -chart_title "pH effect sulphur speciation"
 -axis_titles "pH" "S-speciation (mol)"  "Partial Pressure (atm)"
-axis_scale x_axis      5 14 auto auto
    -axis_scale y_axis      0 0.0016 auto auto
-axis_scale sy_axis     0 0.011 auto auto
    -initial_solutions      true
    -connect_simulations    true
    -plot_concentration_vs  x
  -start
10 graph_x -LA("H+")
20 graph_y  MOL("H2S") MOL("HS-") MOL("S-2") MOL("Fe(HS)2")
```

```
30 graph_sy PR_P("H2S(g)")

USER_GRAPH 3
 -headings head NH3(l) NH4+ NH3(g)
 -chart_title "pH effect ammonia speciation "
 -axis_titles "pH" "NH4-speciation (mol)"  "Partial pressure (atm)"
-axis_scale x_axis      4 14 auto auto
    -axis_scale y_axis      0 0.05 auto auto
-axis_scale sy_axis     0 0.001 auto auto
    -initial_solutions      true
    -connect_simulations    true
    -plot_concentration_vs  x
  -start
10 graph_x -LA("H+")
20 graph_y  MOL("Amm") MOL("AmmH+")
30 graph_sy PR_P("Amm(g)")

USER_GRAPH 4
 -headings head SI("Calcite") SI("Aragonite") SI("FeS(ppt)")
 -chart_title ""
 -axis_titles "pH" "Saturation index"
-axis_scale x_axis      3 14 auto auto
    -axis_scale y_axis      -10 10 auto auto
-initial_solutions      true
    -connect_simulations    true
    -plot_concentration_vs  x
  -start
10 graph_x -LA("H+")
20 graph_y  SI("Calcite") SI("Aragonite") SI("FeS(ppt)")

END
```

SUPPORTING INFORMATION D

Table D. *Summary of effluent characteristics under the Anaerobic (AnMBR) and Micro-aerated (Ma-AnMBR) states.*

	Unit	AnMBR	MA-AnMBR
Chemical oxygen demand (COD) concentration	$mgCOD \cdot L^{-1}$	90.6 ± 4.4	74.6 ± 19.0
Ortho-phosphate concentration	$mgPO_4\text{-}P \cdot L^{-1}$	55.1 ± 0.7	27.6 ± 12.3
Sulphate concentration	$mgSO_4\text{-}S \cdot L^{-1}$	31.9 ± 1.8	23.0 ± 13.0
Ammonium concentration	$mgNH_4\text{-}N \cdot L^{-1}$	547 ± 18	740 ± 106

SUPPORTING INFORMATION E

To further understand the reasons behind the high methane concentration in the biogas, the effect of pH in the dissolved concentrations of total inorganic carbon produced (TIC), HCO_3^-, CO_2, and CO_3^{2-}, was assessed in the PhreeqC model. The total dissolved carbon concentration from these three species increased by around 3% in the MA-AnMBR compared to the AnMBR period. At a pH of 7.6, the MA-AnMBR had a dissolved carbon concentration of 0.42 $g{\cdot}L^{-1}$, while at a pH of 7.4 it was 0.41 $g{\cdot}L^{-1}$ for the AnMBR. The most significant change was observed for carbonic acid, where the model predicted an HCO_3^{-1} concentration of 2.03 $g{\cdot}L^{-1}$ for the MA-AnMBR state and 1.92 $g{\cdot}L^{-1}$ for the AnMBR. Moreover, dissolved CO_2 concentration was also assessed at a neutral pH, and results showed that for both periods, the dissolved CO_2 concentration was 0.35 $g{\cdot}L^{-1}$. This value could also be linked to the predicted increase of CO_2 concentration in the biogas at pH 7, which was around 14 % for both reactor states.

Aside from an increase in the total inorganic carbon produced, the high acid neutralization capacity (ANC) of the AnMBR and MA-AnMBR could be linked to the urea concentration of the feed. A concentration of 1.0 $g{\cdot}L^{-1}$ of urea was added to the synthetic feed. Urea has a molar mass of 60 $g.mol^{-1}$, and each mmol of urea is responsible for producing two meq of ammonia and one of carbon dioxide. At the lab-scale reactor pH, ammonia and carbon dioxide will be mainly in the form of NH_4^+ and HCO_3^-, obtaining a surplus of 17 meq of NH_4^+ cations. Most of the feed's COD content comes from sodium acetate and cellulose, added in concentrations of 2.6 and 1.5 $g{\cdot}L^{-1}$. The first one uses its own sodium as a buffer, and most of the sodium acetate is converted to methane and $NaHCO_3$ [4]. The produced CO_2 from cellulose will be chemically bound with the surplus of NH_4^+ cations coming from urea. This will decrease the CO_2 content in biogas, (increasing the partial content of CH_4), and improve the system buffer capacity, raising

the ANC/TIC ratio. A rise in this ratio increases the HCO_3^- concentration in the liquid and decreases the biogas CO_2 concentration [5]. Therefore, the high methane concentration of the biogas for the AnMBR and MA-AnMBR could be attributed to the feed characteristics, high urea concentration, and CO_2 dissolution into the liquid.

Results of the PhreeqC model regarding methane and carbon dioxide biogas concentrations were further tested with the influent and reactor conditions given by Ozgun, et al. [6], which uses urea as one of the main sources of ammonium. For the lab-scale reactor operated at a pH of 7, Ozgun, et al. obtained a CH_4 concentration of 61 ± 5.7%, while the model predicted a methane concentration of 61%. Moreover, for the same conditions, at a pH of 7.65, the results of the model showed an expected biogas methane concentration of 88%.

SUPPORTING INFORMATION F

Figure F.. *COD balance for the AnMBR and Ma-AnMBR states.*

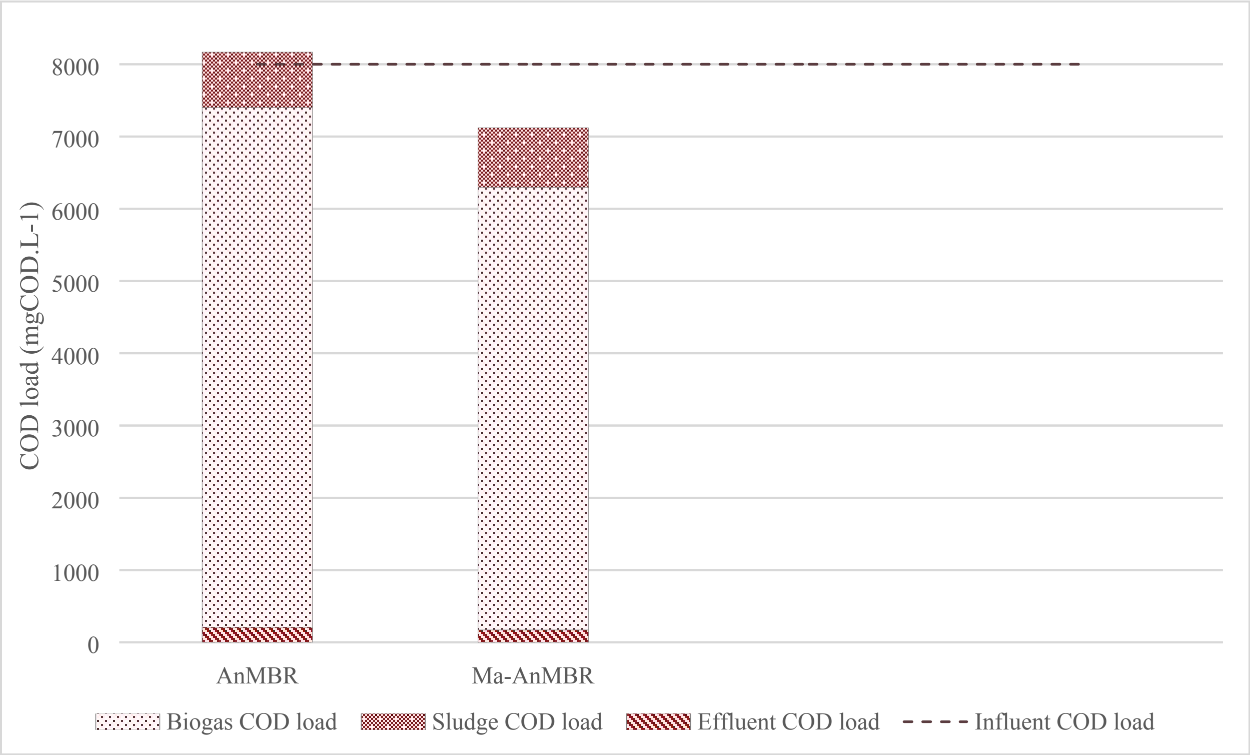

SUPPORTING INFORMATION G

Table G. *P-values from the ANOVA single-factor tests performed for the specific methanogenic activity tests (SMA), when compared to the values of the control tests, with no extra aeration added. SludgeS0 corresponded to sludge from the Anaerobic Membrane Bioreactor (AnMBR) when operated under anaerobic conditions. Inoculums S1 to S3 corresponded to the first three weeks of adaptation to the addition of oxygen to the AnMBR. The last Sludge (S4), corresponded to sludge from the fully adapted Micro-aerated AnMBR. The oxygen supplied to each SMA bottle was calculated as a percentage of the substrate COD load, at 20°C, and considering an air composition of 21% Oxygen and 79% Nitrogen. In bold are the tests that showed significant difference when compared to no aeration of the inoculum.*

Inoculum		3 % of CODin	8 % of CODin	13 % of CODin
S0	Anaerobic	**0.01**	**< 0.01**	**< 0.01**
S1		-	**< 0.01**	-
S2	Adaptation	0.61	**< 0.01**	-
S3		0.39	**< 0.01**	**< 0.01**
S4	MA-AnMBR	0.57	0.36	**0.04**

SUPPORTING INFORMATION H

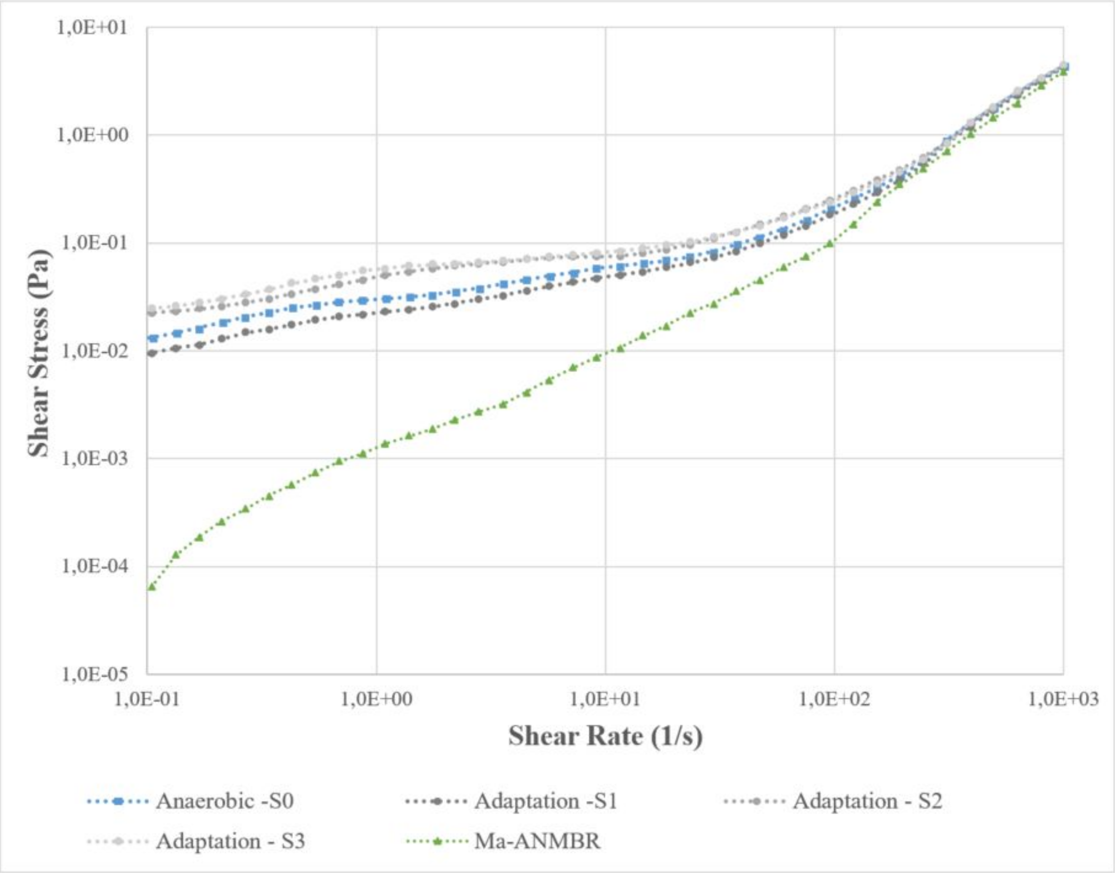

Figure H.*Sludge viscosity curve of the Micro-Aerated AnMBR. . Sludge S0 corresponded to sludge from the Anaerobic Membrane Bioreactor (AnMBR) when was operated under strict anaerobic conditions. Sludge S1 to S3 corresponded to the first three weeks of adaptation to the addition of oxygen to the AnMBR. The last sludge (S4), corresponded to sludge from the fully adapted Micro-aerated AnMBR.*

SUPPORTING INFORMATION G

Methods for microbial community analysis

- **DNA extraction**

Three triplicate sludge samples were taken to perform microbial population analysis, one from the AnMBR state (after 150 days of operation), and two from the MA-AnMBR state (after 370 and 580 days of operation). A homogenised sludge sample of 1.5 mL was transferred into an Eppendorf tube and centrifuged in a micro-centrifuge (Eppendorf, Hamburg, Germany), to extract DNA. Around 50 mg of sludge pellet were added to the extraction tubes from the soil FastDNA spin kit (MP Biomedicals, Irvine, CA, U.S.). DNA extraction was performed following the protocol established by Albertsen, et al. [7]. The concentration of the extracted DNA was measured using a Qubit dsDNA assay kit (Thermo Fisher, Waltham, MA, U.S.A). Finally, the DNA samples were frozen at -25 °C until they were sent for gene amplification.

- **16S rRNA Gene Amplicon Sequencing and Data Analysis**

The 16sRNA gene was selected for amplification and paired-end sequence, in an Ilumina NovaSeq 6000 platform by Novogene (Beijing, China). The primer set chosen to amplify and sequence the hypervariable region V3-V4 was 341F [50'CCTAYGGGRBGCASCAG–3'] and 806R [5'–GGACTACNNGGGTATCTAAT–3']. Subsequently, the Sequence Read Archive (SRA) of the National Center for Biotechnology Information (NCBI) was used for reading the raw data. The gene sequence provided by Novogene was further processed and analysed following the procedure developed by[8] Amir, et al. [8], Toja Ortega, et al. [9].

Results and discussion

To obtain insight in the micro-organisms specific response to the induced oxygen stress DNA analysis was performed for the three samples, the AnMBR state (after 150 days of operation), and two from the MA-AnMBR state (after 370 and 580 days of operation). The two most relatively

abundant taxa for the MA-AnMBR samples were bacteria family *Thermofonsia* or *SBR103* and archaeal family *Methanosaetaceae*, while these taxa were the second and fifth most abundant in the AnMBR samples, respectively. Alpha and beta diversity analyses were conducted to identify further differences between the two reactor states. The diversity within samples (alpha diversity) showed that the phylogenetic distance between taxa in each sample was statistically different (p-value of 0.03), whilst the community evenness within samples was insignificant. Even though the diversity analysis showed a statistical difference between the AnMBR and MA-AnMBR sludge samples, it cannot be linked to differences in AD performance [10].

However, the sequencing of the 16rRNA is not useful to define microorganisms that are responsible for specific processes [11]. For instance, whilst *Methanosaetaceae* family relative abundance increased from 6.2 to 20.7% from the AnMBR to the MA-AnMBR samples (taken after 150 and 370 days respectively), no statistical difference was observed in this group due to the low relative abundance of *Methanosaetaceae* in the Archaea kingdom. While most hydrogenotrophic methanogens are members of the family of *Methanobacteriaceae* [12], aceticlastic methanogens belong to the family of *Methanosaetaceae* [4]. No changes were observed between the sludge samples from the AnMBR and MA-AnMBR in the relative abundance of both families in the Archaea kingdom. *Methanobacteriaceae* represented around 3.5% of the Archaea while the aceticlastic methanogens were around 95.5%. Since hydrogenotrophic and aceticlastic bacteria's relative abundance between the reactor periods did not vary, and SMA results showed no statistical changes in the MA-AnMBR sludge subjected to small aeration, changes in biogas production were not expected.

REFERENCES

[1] A. L. Piaggio, L. A. Soares, M. Balakrishnan, T. Guleria, M. K. de Kreuk, and R. E. Lindeboom, "High suspended solids removal of Indian drain water with a down-scaled Dissolved Air Flotation (DAF) for water recovery. Assessing water-type dependence on process control variables," *Environmental Challenges,* p. 100567, 2022, doi: https://doi.org/10.1016/j.envc.2022.100567.

[2] Wang, Fahey, and Wu, "Dissolved air flotation," in *Physicochemical treatment processes*, vol. vol 3, H. Press Ed., 2005, pp. 431-500.

[3] R. Sander, "Compilation of Henry's law constants (version 4.0) for water as solvent," *Atmospheric Chemistry & Physics,* vol. 15, no. 8, 2015.

[4] J. G. Ferry, "Methane from acetate," *Journal of bacteriology,* vol. 174, no. 17, pp. 5489-5495, 1992, doi: https://doi.org/10.1128/jb.174.17.5489-5495.1992.

[5] R. E. Lindeboom, J. Weijma, and J. B. van Lier, "High-calorific biogas production by selective CO2 retention at autogenerated biogas pressures up to 20 bar," *Environmental science & technology,* vol. 46, no. 3, pp. 1895-1902, 2012, doi: https://doi.org/10.1021/es202633u.

[6] H. Ozgun, M. E. Ersahin, Y. Tao, H. Spanjers, and J. B. van Lier, "Effect of upflow velocity on the effluent membrane fouling potential in membrane coupled upflow anaerobic sludge blanket reactors," *Bioresource technology,* vol. 147, pp. 285-292, 2013, doi: https://doi.org/10.1016/j.biortech.2013.08.039.

[7] M. Albertsen, S. M. Karst, A. S. Ziegler, R. H. Kirkegaard, and P. H. Nielsen, "Back to basics–the influence of DNA extraction and primer choice on phylogenetic analysis of activated sludge communities," *PloS one,* vol. 10, no. 7, p. e0132783, 2015, doi: https://doi.org/10.1371/journal.pone.0132783.

[8] A. Amir *et al.*, "Deblur rapidly resolves single-nucleotide community sequence patterns," *MSystems,* vol. 2, no. 2, pp. 10.1128/msystems. 00191-16, 2017.

[9] S. Toja Ortega, M. Pronk, and M. K. de Kreuk, "Effect of an increased particulate cod load on the aerobic granular sludge process: A full scale study," *Processes,* vol. 9, no. 8, p. 1472, 2021, doi: https://doi.org/10.3390/pr9081472.

[10] Q. Lin, J. De Vrieze, L. Li, X. Fang, and X. Li, "Interconnected versus unconnected microorganisms: Does it matter in anaerobic digestion functioning," *Journal of Environmental Management,* vol. 331, p. 117307, 2023, doi: https://doi.org/10.1016/j.jenvman.2023.117307.

[11] J. De Vrieze, A. J. Pinto, W. T. Sloan, and U. Z. Ijaz, "The active microbial community more accurately reflects the anaerobic digestion process: 16S rRNA (gene) sequencing as a predictive tool," *Microbiome,* vol. 6, no. 1, pp. 1-13, 2018, doi: https://doi.org/10.1186/s40168-018-0449-9.

[12] W. Whitman, D. Boone, Y. Koga, and J. Keswani, "Taxonomy of methanogenic Archaea," in *Bergey's Manual® of Systematic Bacteriology: Volume One The Archaea and the Deeply Branching and Phototrophic Bacteria*: Springer, 2001, pp. 211-213.
